# Expression of vasopressin mRNA in the hypothalamus of individuals with a diagnosis of schizophrenia

**DOI:** 10.1002/brb3.1355

**Published:** 2019-07-13

**Authors:** Johannes R. Busch, Christina Jacobsen, Niels Lynnerup, Jytte Banner, Morten Møller

**Affiliations:** ^1^ Department of Forensic Medicine, Section of Forensic Pathology, Faculty of Health and Medical Sciences University of Copenhagen Copenhagen Denmark; ^2^ Faculty of Health and Medical Sciences, Department of Neuroscience University of Copenhagen Copenhagen Denmark

**Keywords:** mRNA, paraventricular, schizophrenia, vasopressin

## Abstract

**Objective:**

This study investigates the expression of mRNA encoding vasopressin in the hypothalamus of autopsy brains of individuals diagnosed with schizophrenia.

**Methods:**

Ten brains of individuals with schizophrenia and 10 brains from individuals without any disease were examined during autopsy. The hypothalamic block was dissected and immersion fixed in paraformaldehyde, sucrose substituted, frozen, and cut into 20‐µm‐thick coronal cryostat sections. The sections were hybridized with an S‐35‐labeled DNA antisense oligo probe and after washing covered by an X‐ray film. The hybridization signals on the films were transferred to a computer and densitometrically quantified.

**Results:**

The densitometry signals showed a statistically significant lower mRNA expression (53% decrease; *p* = 0.014) in the paraventricular nucleus of the individuals with schizophrenia compared to the controls. In the supraoptic nucleus, the decrease in the group with schizophrenia was 39% compared to the controls, but this decrease was not statistically significant (*p* = 0.194).

**Conclusions:**

Our results show a low expression of mRNA encoding vasopressin in the paraventricular nucleus of the individuals with schizophrenia. We suggest that vasopressin is not directly involved in the pathogenesis of schizophrenia, but might influence schizophrenic symptoms via vasopressin receptors located in the social behavioral neural network in the forebrain.

## 1 SIGNIFICANT OUTCOMES

The expression of mRNA encoding vasopressin in the magnocellular hypothalamic nuclei is decreased in individuals with the diagnosis of schizophrenia.

## 2 LIMITATIONS

The tissue preservation of the brains in a forensic autopsy material varies.

## 3 INTRODUCTION

Schizophrenia is a major psychiatric disorder with an unknown causative pathophysiology (Birnbaum & Weinberger, 2017). Stress could be a factor in the pathophysiology of the disease, and that might involve the endocrine hypothalamic–pituitary–adrenal axis. Thus, stress disorders have been found often to co‐occur with schizophrenia (Seow et al., 2016; Young et al., 2013) although measurements of the stress hormone, cortisol, in plasma and saliva in schizophrenia patients have shown both high, normal, and low levels compared to controls (Bradley & Dinan, 2010). However, several studies measuring the hypothalamic–pituitary–adrenal axis response to psychological stress in schizophrenic patients have demonstrated a blunted adrenocorticotropic hormone (ACTH) and cortisol response to stress (Brenner et al., 2009; Goldman, Gnerlich, & Hussain, 2007; Jansen et al., 1998) indicating an involvement of the hypothalamic–pituitary–adrenal axis in the pathophysiology of schizophrenia.

The nona peptide, vasopressin, is a part of the hypothalamic–pituitary–adrenal axis. This hormone is located in magnocellular neurons in the human hypothalamic paraventricular nucleus of the hypothalamus (Møller et al., 2018; Saper, 2012) in which parvocellular neurons, containing corticotropin‐releasing hormone (CRH), also are located. Although the main function of vasopressin, also called the antidiuretic hormone (ADH), is the regulation of water re‐absorption in the kidney, via projections of axons to the posterior pituitary lobe and release of the hormone to the vascular system, vasopressin is also involved in regulation of cortisol from the suprarenal glands (Aguilera & Rabadan‐Diehl, 2000). Thus, vasopressin released via the portal capillaries of the median eminence to the anterior lobe of the pituitary stimulates the pituitary ACTH secretion by potentiating the stimulatory effects of CRH on the pituitary ACTH secreting cells (Aguilera & Rabadan‐Diehl, 2000; Naughton, Dinan, & Scott, 2014).

In addition to the involvement in water regulation and stress, vasopressin modulates social interactions (Donaldson & Young, 2008), and intranasal administration of the vasopressin analogue desmopressin has shown a positive effect on negative schizophrenic symptoms (Brambilla et al., 1989; Hosseini et al., 2014).

Several studies have shown alterations in vasopressin levels in plasma and cerebrospinal fluid in schizophrenic patients (Bradley & Dinan, 2010; Jobst et al., 2014). Although some earlier studies have shown elevated level of vasopressin, most studies have shown a lower level in schizophrenia patients compared to human controls (Frederiksen, Ekman, Gottfries, Widerlöv, & Jonsson, 1991; Rubin et al., 2013, 2014).

Modern molecular biological methods have made it possible to determine the amount of mRNA encoding for vasopressin in the hypothalamic neurons of the hypothalamus. We have in this study, by use of quantitative radioactive in situ hybridization, compared the intensity of the mRNA signal in sections of the hypothalamus from brains removed during autopsies of individuals with a diagnosis of schizophrenia to the signal intensity in a series of control brains. Our results demonstrate a lower mRNA expression level in the brains of the schizophrenic patients compared to the levels in the controls.

### 3.1 Aims of the study

1. In a series of autopsy brains to determine the expression levels of mRNA encoding vasopressin in the hypothalamus of individuals with the diagnosis of schizophrenia.
2. To compare the expression in the brains of schizophrenic individuals with the expression in a control series.

Our hypothesis is that vasopressin is not directly involved in the pathogenesis of schizophrenia, but might influence schizophrenic symptoms.

## 4 MATERIALS AND METHODS

### 4.1 Removal of specimens and fixation

Brains used in this study were examined during autopsies performed at the Department of Forensic Medicine, University of Copenhagen, as a part of the Danish National SURVIVE study http://retsmedicin.ku.dk/english/research/surviveprojects/. Ten of the brains were from individuals with a diagnosis of schizophrenia and 10 additional brains were from persons without any brain pathology or disease and served as controls (for detailed subject data, see Table 1). Family members were asked for permission to use the tissues for scientific investigations and publishing of data. The study conforms to recognized standards, for example: Declaration of Helsinki (WMA General Assembly, Seoul, Korea, October 2008). The SURVIVE study was approved by the Danish National Committee on Research Ethics (reference number: 1305373) and the Danish Data Protection Agency (reference number: SUND‐2016‐16).

**Table 1:** Clinicopathological data of the cases studied

Age	Minimal postmortem interval (days)	Maximal postmortem interval (days)	Gender	Schizophrenia diagnosis	Cause of death
43	6,5	7,5	Male	No	Poisoning
56	6,1	7,3	Female	No	Hypovolemia
58	4,6	4,9	Male	No	Heart failure
48	0,9	1,0	Female	No	Asphyxia
30	4,2	11,0	Male	No	Ketoacidosis
72	4,2	6,6	Female	No	Pulmon.embolism
21	5,3	6,0	Male	No	Poisoning
65	3,4	4,3	Female	No	Asphyxia
60	5,4	6,1	Male	No	Poisoning
71	2,7	2,8	Male	No	Poisoning
58	2,4	3,2	Female	Yes	Poisoning
58	6,1	6,2	Female	Yes	Asphyxia
41	5,6	6,1	Male	Yes	Poisoning
49	7,5	8,1	Male	Yes	Undetermined
31	11,1	11,1	Female	Yes	Asphyxia
45	17,1	17,1	Male	Yes	Poisoning
59	1,4	2,0	Female	Yes	Poisoning
45	3,5	3,6	Male	Yes	Heart failure
42	4,1	4,1	Male	Yes	Pulmon.embolism
67	4,5	5,1	Female	Yes	Heart failure

During examination of the brain, a tissue block containing the hypothalamus was dissected from the ventral part of the forebrain. The rostral boundary was a coronal section 0.75 cm rostral to the optic chiasm, and the caudal boundary was just behind the mammillary bodies. Laterally, two sagittal sections were made through the optic tracts 2 cm lateral to the midline (third ventricle). The hypothalamus was finally isolated from the dorsal part of the diencephalon with a horizontal cut just below the thalamic nuclear complex above the hypothalamic sulcus.

The tissue blocks were fixed in 4% formalin for 2–4 months and after fixation cryoprotected in 25% sucrose in distilled water for 7 days. The tissues blocks of the hypothalamus were then frozen in crushed carbon dioxide and stored at −80°C. Twenty‐micrometer‐thick serial coronal sections through the supraoptic area were cut in a Leitz cryostat and mounted on Super Frost^®^ glass slides (Menzel). Every 10th section was Nissl counter stained. Four sections of the coronal series were selected for in situ hybridization. The first rostral section was cut through the middle of the optic chiasm, where the supraoptic nucleus is large and exhibiting a triangular shape. The next three sections used for in situ hybridizations were located 100, 200, and 300 µm caudal to first rostral section. The sections selected for in situ hybridizations were stored at −80°C.

### 4.2 Radiochemical in situ hybridization for histological detection of vasopressin transcripts

Tissue sections were thawed and washed 2 × 1 min in PBS. This was followed by acetylation in 0.25% acetic anhydride (diluted in 0.1 m triethanolamine and 0.9% NaCl) for 10 min. The sections were then dehydrated in a graded series of ethanols and delipidated in 100% chloroform, followed by partial rehydration in 95% ethanol. A 48‐mer antisense DNA probe corresponding to bases 1960–2007 of human vasopressin mRNA (XM_011529267; 5′‐gcaaggccccggccggcccgtccagctgcgtggcgttgctccggtcgc‐3′) was diluted in DEPC‐treated water to a concentration of 5 pmol/µl. Five microliters of the probe was then labeled with [^35^S]dATP (code nr. NEG034H250UC; Perkin Elmer) by use of terminal transferase (code nr. 3333566001; Roche) to a specific activity of 1 × 10^18^ dpm/mol. The labeled probe was diluted in a hybridization buffer (5 µl labeled probe/ml hybridization buffer) consisting of 50% (v/v) formamide, 4 × SSC (SSC: 150 mm NaCl, 15 mm sodium citrate, pH 7.0), 1 × Denhardt solution (0.02% bovine serum albumin, 0.02% polyvinylpyrrolidone, 0.02% ficoll), 10% (w/v) dextran sulfate, 10 mm dithiothreitol, 0.5 mg/ml salmon sperm DNA, and 0.5 mg/ml yeast tRNA. The sections were hybridized in a humid chamber overnight at 37°C. After hybridization, the slides were washed in 1 × SSC for 4 × 15 min at 55°C, 2 × 30 min at room temperature and rinsed in deionized water.

The sections were dried and exposed to an X‐ray film for 19 days. After development of the films, the images of the sections were transferred to a computer and quantified by use of the software ScionImage (Wayne Rasband, National Institutes of Health). Optical densities were converted to dpm/mg tissue by using simultaneously exposed ^14^C‐standards calibrated by comparison with ^35^S‐tissue paste standards. Signals from the supraoptic and paraventricular nuclei on both sides of the hypothalamus were obtained.

### 4.3 Controls

In situ hybridizations were done on parallel sections of the same hypothalamic blocs with sense and antisense probes against vasopressin and corticotropin‐releasing hormone (CRH).

### 4.4 Statistical data analysis

The densities of the vasopressin expression signals on the X‐ray films in the paraventricular and the supraoptic nuclei were analyzed by use of GraphPad Prism 7.0. The signals from the individuals with a schizophrenia diagnosis and the control brains were compared in the paraventricular nuclei and also in the supraoptic nuclei by use of the unpaired Student's *t* test. A *p*‐value below 0.05 was considered statistical significant.

## 5 RESULTS

### 5.1 Morphology of the paraventricular and supraoptic nuclei in the supraoptic region

**Figure 1:**
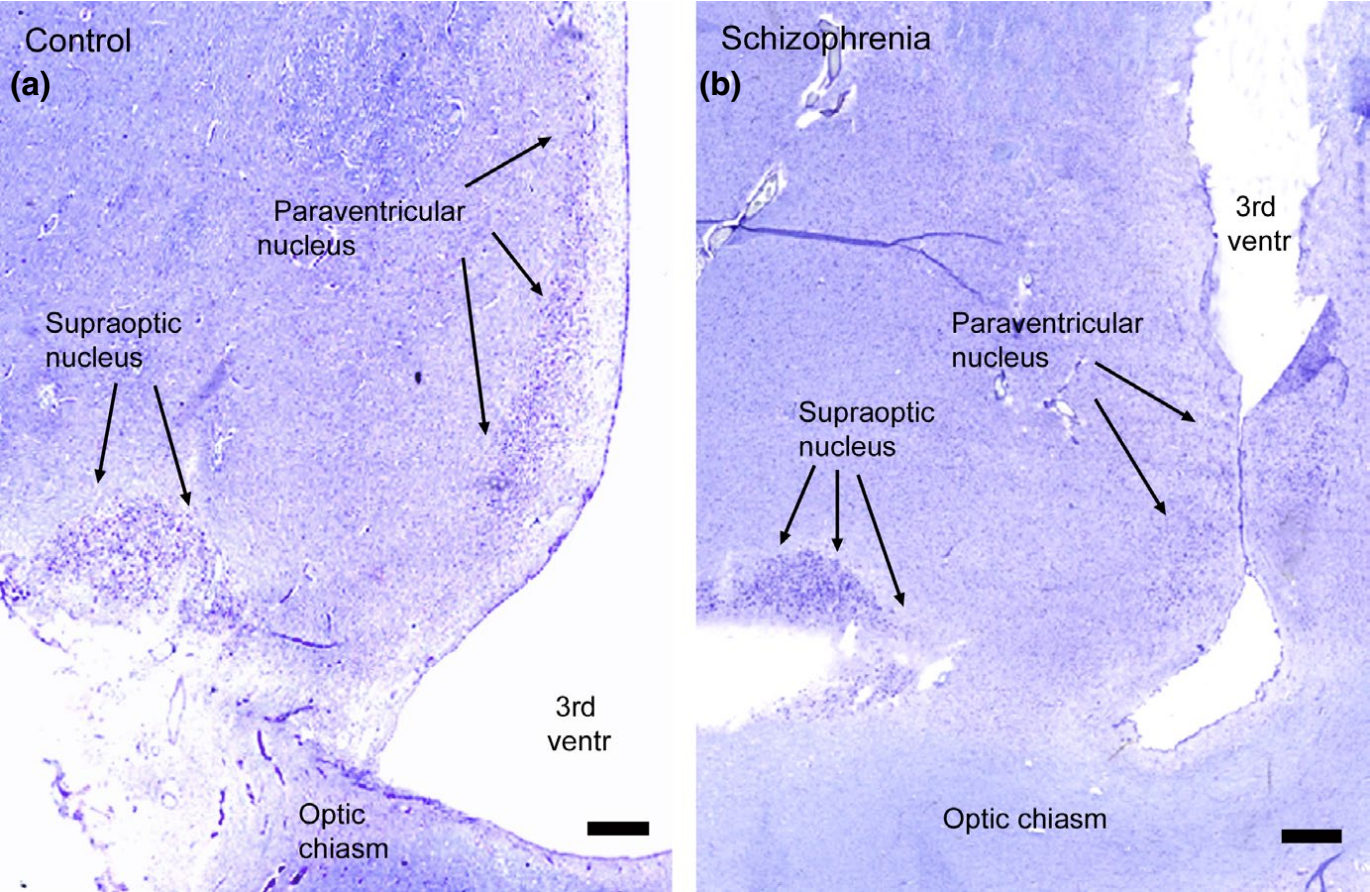
Nissl counterstaining of the sections used for in situ hybridization for mRNA encoding vasopressin, the expression signals of which are shown in Figure 2. (a) Section from a control brain. (b) Section from the brain of an individual with the diagnosis of schizophrenia. 3rd ventr, third ventricle. Bars = 1 mm

In coronal sections, the bilateral magnocellular paraventricular nuclei are located just lateral to the third ventricle exhibiting an ovoid shape along the ventricle (Figure 1a,b). The large supraoptic nuclei are located ventrally in the hypothalamus just above the inferior surface. At the level of the optic chiasm, the nuclei exhibit a triangular shape just lateral to the chiasm (Figure 1a,b), but possess a long and thin tail region stretching along the optic tracts.

### 5.2 Densitometry of the vasopressin expression signals in the paraventricular and supraoptic nuclei in the supraoptic region

**Figure 2:**
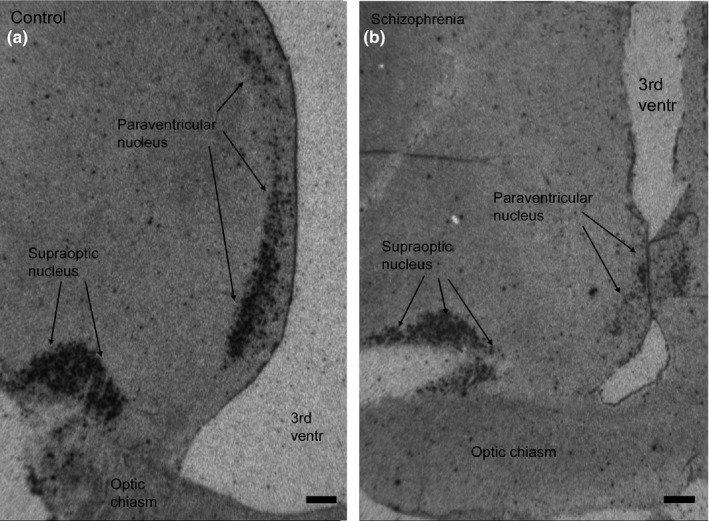
X‐ray images of coronal sections through the supraoptic area of the hypothalamus hybridized with an antisense oligo probe against mRNA encoding vasopressin. On the left (a) a section from a control brain and to the right (b) a section from an individual with a diagnosis of schizophrenia. Expression signals are seen above the nerve cell bodies in the supraoptic and paraventricular nuclei in both sections, but the intensity is lower in the section from the individual with the diagnosis of schizophrenia. 3rd ventr, third ventricle. Bars = 1 mm

**Figure 3:**
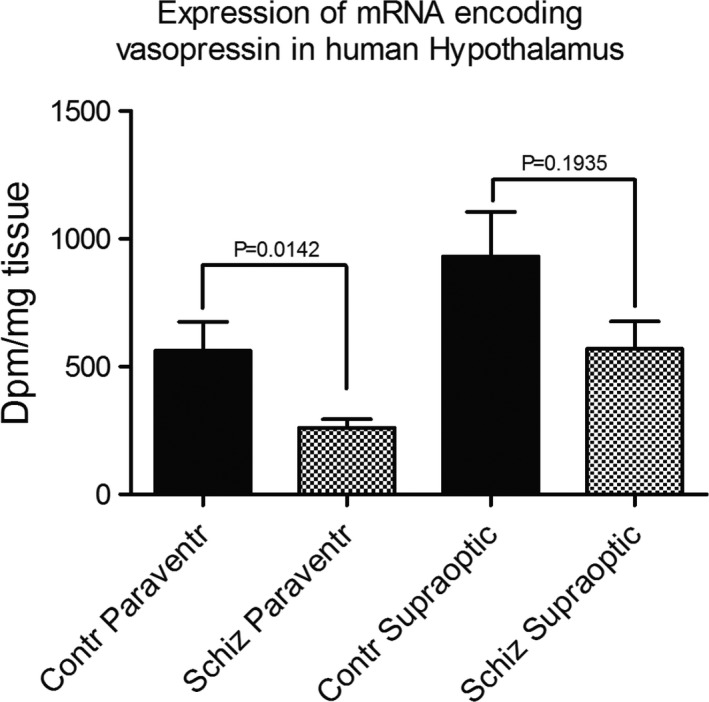
Bar graph of the densitometry determinations of the vasopressin signals in the hypothalamic sections of control person and a person with the diagnosis of schizophrenia. Ordinate shows disintegrations per minutes per mg tissue. The columns on the abscissa show the signal means of the sections from the paraventricular nuclei of control persons (*Control paraventr*) and signal means of the sections from individuals with the diagnosis of schizophrenia (*Schiz paraventr*); further, signal means of supraoptic control sections (*Control supraoptic*) and supraoptic sections from individuals with the diagnosis of schizophrenia (*Schiz supraoptic*). *n* = 10. *p*‐values obtained by use of the unpaired Student's *t* test are shown above the bars. The line above average is the standard error of mean (*SEM*)

Both the supraoptic and paraventricular nuclei showed strong radioactive signals on the developed X‐ray films, indicating a high expression of vasopressin in these nuclei (Figure 2a,b). The signals were located above the magnocellular perikarya of the paraventricular and supraoptic nuclei. The intensity of the signals was lower in the sections of the individuals with the diagnosis of schizophrenia compared to the sections from the controls. This was confirmed in the densitometry measurements of the signals on the films showing a statistical significant lower signal (53% decrease) in the paraventricular nucleus of the schizophrenic patients compared to the controls (Figure 3). In the supraoptic nucleus, the decrease in the schizophrenic patients was 39% compared to the controls, but this decrease was not statistically significant (Figure 3).

## 6 DISCUSSION

Many reports regarding vasopressin and schizophrenia have been published but a clear picture of how this hormone is involved in the disease has not been obtained, and the results obtained are still conflicting.

Thus, changes in the amount of vasopressin in the brain of schizophrenic patients were first reported in investigations 30 years ago, in which a decrease in vasopressin in the temporal cortex was observed, but a reduction in the hypothalamus was not detected (Frederiksen et al., 1991).

Studies of the plasma levels of vasopressin in schizophrenic patients have been giving conflicting results. Most studies have reported a decrease in vasopressin levels of schizophrenic patients compared to controls (Goldman et al., 2007; Jobst et al., 2014; Linkowski, Geenan, Kerkhofs, Mendlewicz, & Legros, 1984; Ryan, Sharifi, Condren, & Thakore, 2004). However, in a recent study, an increase in plasma concentration of vasopressin in schizophrenic patients compared to controls was reported (Guzel et al., 2018). Also, in a recent study by Aydin and coworkers no difference in plasma vasopressin in schizophrenic patients was detected (Aydın, Lysaker, Balıkçı, Ünal‐Aydın, & Esen‐Danacı, 2018). The time of day when the blood samples were obtained was rarely given in above quoted studies. However, because neither the CSF nor plasma levels show a significant circadian rhythm in adult humans (Barreca et al., 1988; Mahler et al., 2013), the difference in plasma levels obtained in these studies cannot be due to variations in circadian time between study groups. We have in this study not been able to obtain information about medication, including neuroleptics, taken by the controls and schizophrenic patients. This would have been of interest due to influence of dopamine agonists and antagonists on vasopressin secretion (Locatelli, Bresciani, Tamiazzo, & Torsello, 2010). Forensic toxicology showed the presence of antipsychotics in two in the control group and four in the schizophrenic group. Treatment with neuroleptics, which are dopamine antagonist, should have resulted in a decreased vasopressin release and higher vasopressin immunoreactivity in the neurons of the paraventricular nucleus. However, the change in the expression of mRNA encoding vasopressin after neuroleptics is uncertain.

The decrease in hypothalamic expression of mRNA encoding vasopressin in individuals with a diagnosis of schizophrenia found in this study is in accord with the majority of earlier studies. Thus, already investigations made by Gerber, who applied Gomori's trichrome staining to hypothalamic sections, observed a reduced cellular content of neurosecretory granules in neurons of neuroleptic‐treated individuals with schizophrenia (Gerber, 1965). Later using antibodies against neurophysin I and neurophysin II, the carrier proteins for vasopressin (neurophysin II) and oxytocin (neurophysin I), a reduction of neurophysin immunoreactive neurons in the paraventricular nucleus was observed in schizophrenic patients compared to controls (Bernstein, Dobrowolny, Bogerts, Keilhoff, & Steiner, 2018; Mai, Berger, & Sofroniew, 1993).

Our observation of a decreased expression of mRNA encoding vasopressin in only the paraventricular nucleus and not in the supraoptic nucleus is also in accord with most previous studies. Thus, in the above quoted study by Mai et al. (1993), these authors did not find a reduction in neurophysin staining in the supraoptic nucleus of the schizophrenic patients compared to controls. Further, in a combined immunohistochemical and in situ hybridization study of vasopressin in the supraoptic nucleus, it was not possible to detect any difference in vasopressin expression and vasopressin mRNA expression in this nucleus in schizophrenic patients compared to controls (Panayotacopoulou, Malidelis, Heerikhuize, Unmehopa, & Swaab, 2005).

An important question related to this study is the stability of mRNA encoding vasopressin in the human autopsy brains, used in our investigation, as a function of postmortem time. The effect of the postmortem interval and preservation of mRNA in autopsy materials has been investigated in several studies (Birdsill, Walker, Lue, Sue, & Beach, 2011; Durrenberger et al., 2010; Stan et al., 2006; Trabzuni et al., 2011; White et al., 2018). In several of these studies, it has been difficult to verify a direct relationship between the postmortem time of the brain tissue and mRNA degradation. However, in the paper by Birdsill et al. (2011) it is shown that RNA degrades progressively with increasing postmortem time but with high between‐subject variability. This might be due to variable agonal conditions and especially the temperature environment (White et al., 2018).

In our immunohistochemical investigations of the accessory magnocellular neurosecretory system of the human hypothalamus (Møller et al., 2018), we found no correlation between the postmortem time and the immunoreactivity in our sections. An explanation for that several of our autopsy brains with long postmortem time has preserved protein, and mRNA might be due to the fact that the dead bodies in our study were kept cold in the morgue during most of the postmortem time, and after removal of the brains, these were immediate fixed by immersion in cold formalin.

**Figure 4:**
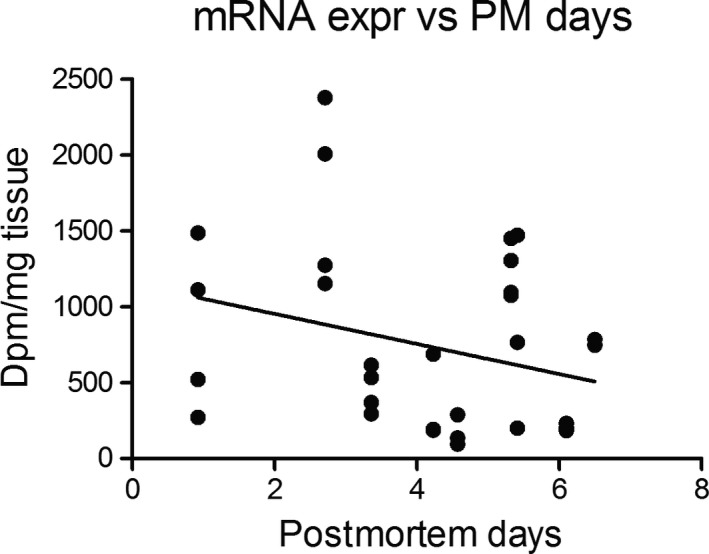
Linear regression analysis of postmortem days versus expression of mRNA encoding vasopressin in the paraventricular nucleus of hypothalamus of control brains. The expression signal shown on the ordinate is disintegrations per minute per mg tissue obtained in sections of the control brains. *R* square = 0.08095, *p* = 0.1145

It has not been possible in our histological study to do reverse transcription‐polymerase chain reactions (qRT‐PCR) on the same tissue. However, we have performed a linear regression analysis of the expression signals in the paraventricular nucleus of the control brains as a function of postmortem time (Figure 4). Although the regression line of Figure 4 shows a decline of mRNA expression as a function of postmortem days, the slope deviation is not significant from zero (*p* = 0.115).

Regarding a possible physiological influence of vasopressin on brain function, it must be kept in mind that effector molecule is the peptide and not the mRNAs encoding the peptides. This raises the question of whether mRNA expression is correlated with the peptide or protein expression. Recent advances in next‐generation DNA sequencing and proteomics have provided an ability to study mRNA and protein abundances in cells. By use of these techniques in mammalian cells, 30%–40% of the variance in protein abundance can be explained by mRNA abundance (Vogel & Marcotte, 2012). This problem has also been investigated in human postmortem hypothalamus. Thus, a combined immunohistochemical and in situ hybridization study showed that the number of cell bodies immunoreactive to tyrosine hydroxylase, the rate‐limiting enzyme in the catecholamine synthesis, and mRNA encoding tyrosine hydroxylase was well correlated (Panayotacopoulou et al., 2005). We have in our material of the brains of deceased with schizophrenia investigated the expression of proteins by use of antibodies against both vasopressin and neurophysin. Both hormones are present in neurons in the paraventricular and supraoptic nuclei. However, due to the variations in the immunohistochemical staining in our material, we did not perform a quantification of these expressions. This indicates that the immunohistochemical technique is less sensitive than the radioactive in situ hybridization (Kim et al., 2009), also in postmortem brain tissue.

Schizophrenia is a complex neurobiological disorder in which genetic, developmental, and environmental factors have been identified to play a role (Birnbaum & Weinberger, 2017; Keller, 2018). The final pathophysiology involves a dysregulation of dopaminergic, glutamatergic, GABAergic, and cholinergic neurotransmitter systems and their interactions (Siever & Davis, 2004).

How the low vasopressin expression in the paraventricular nucleus in schizophrenic patients is involved in the disease is not clear. The low expression of mRNA encoding vasopressin in the classical magnocellular hypothalamic nuclei indicates that stress involving these nuclei is not an important factor in the disease. We have also tried in situ hybridization for mRNA encoding for corticotropin‐releasing hormone, but have not obtained satisfactory signals for this mRNA.

Although vasopressin might not be directly a factor in the pathogenesis of the schizophrenia, the hormone might contribute to the symptomology of the disease. Thus, vasopressin in humans is capable of influencing a wide variety of behavioral traits (Bachner‐Melman & Ebstein, 2014; Landgraf, Wotjak, Neumann, & Engelmann, 1998) for example, traits associated with arousal, vigilance, and defensive behaviors, symptoms often seen in schizophrenia (Reser, 2007). This possible function of vasopressin in humans is supported by numerous animal studies showing the involvement of vasopressin in social recognition, communication, and aggression (Albers, 2012). There is considerable evidence that many of the effects of vasopressin on social behavior are mediated by the vasopressin 1a and 1b receptor subtypes (Stevenson & Caldwell, 2012). These receptors are extensively expressed throughout the so‐called social behavior neural network in the forebrain, a network composed of neural groups located in the lateral septum, amygdala, and the preoptic area in rodent and primate species (Newman, 1999). These areas also show high vasopressin binding in the human brain (Loup, Tribollet, Dubois‐Dauphin, & Dreifuss, 1999).

Finally, vasopressin and vasopressin analogs applied intranasal to schizophrenic patients resulted in an improvement of both the patient's memory functions (Geng et al., 2017) and the negative symptoms (Jobst et al., 2014).

Summarizing, this paper shows a reduced expression of mRNA encoding vasopressin in the paraventricular nucleus, in brains of individuals with the diagnosis schizophrenia, compared to control brains. No change was seen in the supraoptic nucleus of the hypothalamus. Vasopressin is probably not directly involved in the pathogenesis of schizophrenia, but might influence schizophrenic symptoms.

## Data Availability

The data that support the findings of this study are available from the corresponding author upon reasonable request.
